# Elemental diet and the nutritional treatment of Crohn’s disease

**Published:** 2015

**Authors:** John Hunter

**Affiliations:** *Honorary Consultant Physician Addenbrookes Hospital Cambridge, UK*

The study by Rostami and Al Dulami in the present edition of the journal adds a further indication for the use of Elemental diet in Inflammatory Bowel Disease, namely the treatment of a high output stoma (1). The authors stress the value of such treatment in a range of disorders, but its main role remains in the treatment of Crohn’s disease.

The value of enteral feeds in Crohn’s disease was first reported over 30 years ago (2,3).  However, despite general agreement of the effectiveness of enteral feeding and the difficulties generally encountered in managing Crohn’s disease, diet is still infrequently used.  This may partly be an effect of the intense publicity given to treatment with biological agents in recent years, but it also reflects a number of other factors. 

Perhaps the most important of these has been failure to understand the mechanisms by which enteral feeding works.  Crohn’s disease has long been a puzzle to gastroenterologists but the clouds are gradually clearing and studies on the effects of enteral feed have helped considerably. 

Although in health we live in peace with our microflora, the colonic microflora is abnormal in Crohn’s disease (4). This may lead to production of toxic chemicals such as alcohols, aldehydes and the ethyl esters of fatty acids (5).  It is believed that this is the reason for the loss of normal immune tolerance to the gut flora in Crohn’s disease, which results in the coating of faecal bacteria by immunoglobulin (6-8).  Elemental diet has been shown to reduce the production of bacterial metabolites (5) within 2 weeks and significantly to reduce bacterial coating with immunoglobulin (8).

Thus enteral feeds act directly on the microbiota.  Although many still assume that their effects must be related to food allergy, this is not the case and manifestations of genuine IgE and IgG food allergies do not apply.

Overall, the results of enteral feeding are excellent with 80-100% of compliant patients going into remission within 2-3 weeks.  The efficacy of these feeds appears to be more closely related to the amount of energy coming from long chain triglyceride rather than the presentation of nitrogen (9). Such results compare favourably with those achieved by treatment of Crohn’s disease with immunosuppression.  Why then, is the nutritional approach so infrequently used?

Clearly, patient compliance is one factor.  In most studies approximately 25% are unwilling to restrict their nutritional intake to a liquid feed for as long as 2-3 weeks and 5% in our experience find the taste unpalatable.  This accounts for the ‘failure’ of enteral feeding to appear superior to corticosteroids in intention to treat studies (10).  However, most patients who have experienced difficulties with pharmacological treatments are willing to accept the inconvenience involved.

There are also disagreements about how patients should be managed when they achieve remission.  The reintroduction of normal foodstuffs is still controversial, but the value of detecting specific food intolerances and building up a personalised exclusion diet for long term remission is well documented (11-13). A diet low in fat and fibre (LOFFLEX) has been shown to be highly effective with nearly 60% of patients in remission after 2 years (14).  Foods involved vary from patient to patient but may include cereals such as wheat, maize and oats, dairy products, pork, onions and yeast. The process of food testing involves trial and error and requires patience. It is therefore essential that dietitians are available to ensure diets remain nutritionally adequate. 

Such specialist support is not available in all centres and some gastroenterologists lack confidence in managing nutrition problems. This may deter many from trying this approach, despite its proven lack of side effects such as osteoporosis and safety in pregnancy (15, 16). However, surely it must be available in tertiary centres dealing with complex refractory cases of Crohn’s disease.

## Note

Please see the pages: 71-76
